# Retrograde drilling for ankle joint osteochondral lesions: a systematic review

**DOI:** 10.1186/s10195-023-00716-4

**Published:** 2023-07-26

**Authors:** Elena Artioli, Antonio Mazzotti, Simone Gerardi, Alberto Arceri, Francesca Barile, Marco Manzetti, Giovanni Viroli, Alberto Ruffilli, Cesare Faldini

**Affiliations:** 1 IRCCS Istituto Ortopedico Rizzoli, 1st Orthopaedics and Traumatology Clinic—University of Bologna, Via Giulio Cesare Pupilli 1, 40136 Bologna, Italy; 2 Department of Biomedical and Neuromotor Sciences, University of Bologna, 40123 Bologna, Italy

**Keywords:** Drilling, Osteochondral lesions, Ankle, Cartilage

## Abstract

**Background:**

Extensive literature exists about the treatment of ankle osteochondral lesions, but there is no specific review of retrograde drilling, despite its common application. Indications for retrograde drilling are still few and are far from clear, and some evolutions of the technique have recently occurred. The aim of this review is to provide an update on actual applications and techniques of retrograde drilling for ankle osteochondral lesions.

**Methods:**

A systematic review was carried out according to the 2020 PRISMA guidelines. The PubMed and Embase databases were searched in June 2023. The search string focused on studies related to retrograde drilling in the treatment of ankle osteochondral lesions.

**Results:**

Twenty-one articles for a total of 271 ankles were included in this review. The mean length of the treated lesions was 11.4 mm. Different navigation systems were used, with fluoroscopy the most commonly used. Various adjuvants were employed after drilling, with bone graft the most commonly applied. In most cases, postoperative patient satisfaction and symptom relief were reported, and no complications occurred. Retrograde drilling was found to be suitable for the treatment of subchondral cysts with intact cartilage or small lesions. Some modifications to the original technique may allow surgical indications to be extended to more complex cases.

**Conclusions:**

Middle-term results of retrograde drilling showed postoperative satisfaction and symptom relief with both original and modified techniques. Additional research is required to investigate the long-term results.

*Level of evidence*: IV.

*Trial registration*: This systematic review was registered on PROSPERO (id number: CRD42022371128).

## Introduction

Osteochondral lesions are defects of the subchondral bone and the overlying cartilage. Active and young patients are the most commonly affected by this disorder, which causes chronic pain, swelling, and stiffness [1, 2] and is supposed to occur in up to 50% of ankle sprain cases [3]. When conservative treatment fails, surgical management is indicated [1, 4]. Among the various surgical techniques available, drilling represents a common and widespread procedure because of its simplicity and cost-effectiveness. Two variants of drilling can be distinguished based on the direction of the drill toward the lesion: anterograde or retrograde. Anterograde or transmalleolar drilling was described by Kumai et al. in 1999 [5] and consists of the insertion of a K-wire from the medial malleolus directly to the lesion, passing through the intact cartilage [5]. Instead, retrograde drilling (RD) allows the cartilage layer to be preserved by reaching the lesion from behind. RD has the advantage of not damaging the cartilage, so it is particularly indicated in the treatment of subchondral cysts or any osteochondral lesions with an intact and stable cartilage surface [1].

Despite its wide application, current evidence on the indications for and outcomes of RD in the treatment of osteochondral lesions of the ankle is limited, and a comprehensive review is missing. Moreover, in the last few years there has been some evolution of the original technique [6–8]. Since RD has provided better results compared to anterograde drilling [9], and the latter is being progressively replaced by microfractures that do not cause heat damage, this review focuses only on RD.

The aim of this systematic review is therefore to provide an update about actual applications, techniques, and outcomes of RD for ankle osteochondral lesions.

## Materials and methods

### Eligibility criteria

All articles written in English on the treatment of osteochondral lesions of the talus and distal tibia through RD were included in this review. Exclusion criteria were anterograde drilling; RD associated with microfractures, osteochondral transplant, debridement; surgical procedures other than RD (such as microfractures, autologous matrix-induced chondrogenesis, osteochondral autograft or allograft transplant, mosaicplasty, matrix-assisted autologous chondrocyte transplantion); osteochondral lesion sites other than the distal tibia or talus; studies reporting data that do not distinguish among the compared surgical procedures; studies involving cadaveric or animal specimens; single-case reports, editor commentaries, letters to the editor, reviews, and articles not written in English.

No exclusion criteria were applied based on age, sample size, and follow-up.

### Search strategy

This systematic review was conducted according to PRISMA (Preferred Reporting Items for Systematic Reviews and Meta-analyses) guidelines [10] and registered on PROSPERO (id number: CRD42022371128).

The PICO algorithm was established as follows:

- P (problem): osteochondral lesions of the talus and distal tibia
- I (intervention): RD
- C (comparison): no comparison group
- O (outcomes): clinical scores, patient satisfaction, and complications

A comprehensive literary search was run across the PubMed and Embase databases in June 2023. A combination of the following keywords and the Boolean indicator AND was used: drilling, osteochondral, lesion. Results of the database search are reported in Table 1.

**Table 1:** Summary of the results of the database search

PubMed: (drilling) AND (osteochondral) AND (lesion)	Embase: (drilling) AND (osteochondral) AND (lesion)
No. of articles: 347	No. of articles: 258
No. of articles after duplicate removed: 433	

### Selection and data collection

After duplicate removal, two authors (EA, SG) independently reviewed all the articles by title and abstract to select those eligible for inclusion based on the aforementioned inclusion and exclusion criteria. The full texts of the retrieved articles were accessed. The bibliographies of the eligible studies were carefully examined to identify additional articles of interest. In the case of uncertainty, the senior author made the final decision.

### Data items

Full texts of the retrieved articles were carefully examined to extract the following data. The characteristics of each study—in terms of authors, year of publication, study type, and level of evidence (LOE) according to the Oxford Level of Evidence scale—were noted. Population data were collected, considering the number of patients, number of ankles, sex of patients, mean age at surgery, mean follow-up, and location and size of the osteochondral lesions. Indications consisting of parameters to consider surgery, classification systems, and grades of the lesions. Data concerning the surgical procedure such as the navigation system and adjuvants to the original technique were collected. Clinical outcomes were obtained through the American Orthopedic Foot and Ankle Society's ankle-hindfoot scale (AOFAS), the Visual Analog Scale (VAS), and clinical satisfaction. Intraoperative and postoperative complications were noted.

### Assessment of risk of bias and quality of recommendation

Quality assessments of the included studies were conducted through ROBINS-I for non-randomized studies [11]. The following factors pertaining to risk of bias were evaluated: confounding, selection, classification of interventions, deviation from intended intervention, missing data, measurement of outcomes, and selection of the reported results.

The quality of evidence was assessed utilizing the Grading of Recommendations Assessment, Development and Evaluation (GRADE) framework.

### Data synthesis

Categorical variables were reported as the frequency and/or percentage, while continuous variables were reported as the mean value and its range. Given the differences in reporting patient satisfaction, the following dichotomic classification of the results was needed: “good” and “excellent” were grouped as “satisfied,” and “fair” and “poor” as “unsatisfied”. Data collection was performed using Microsoft Excel (Microsoft Corporation, Redmond, WA) for Windows 11.

## Results

### Search results

**Fig. 1:**
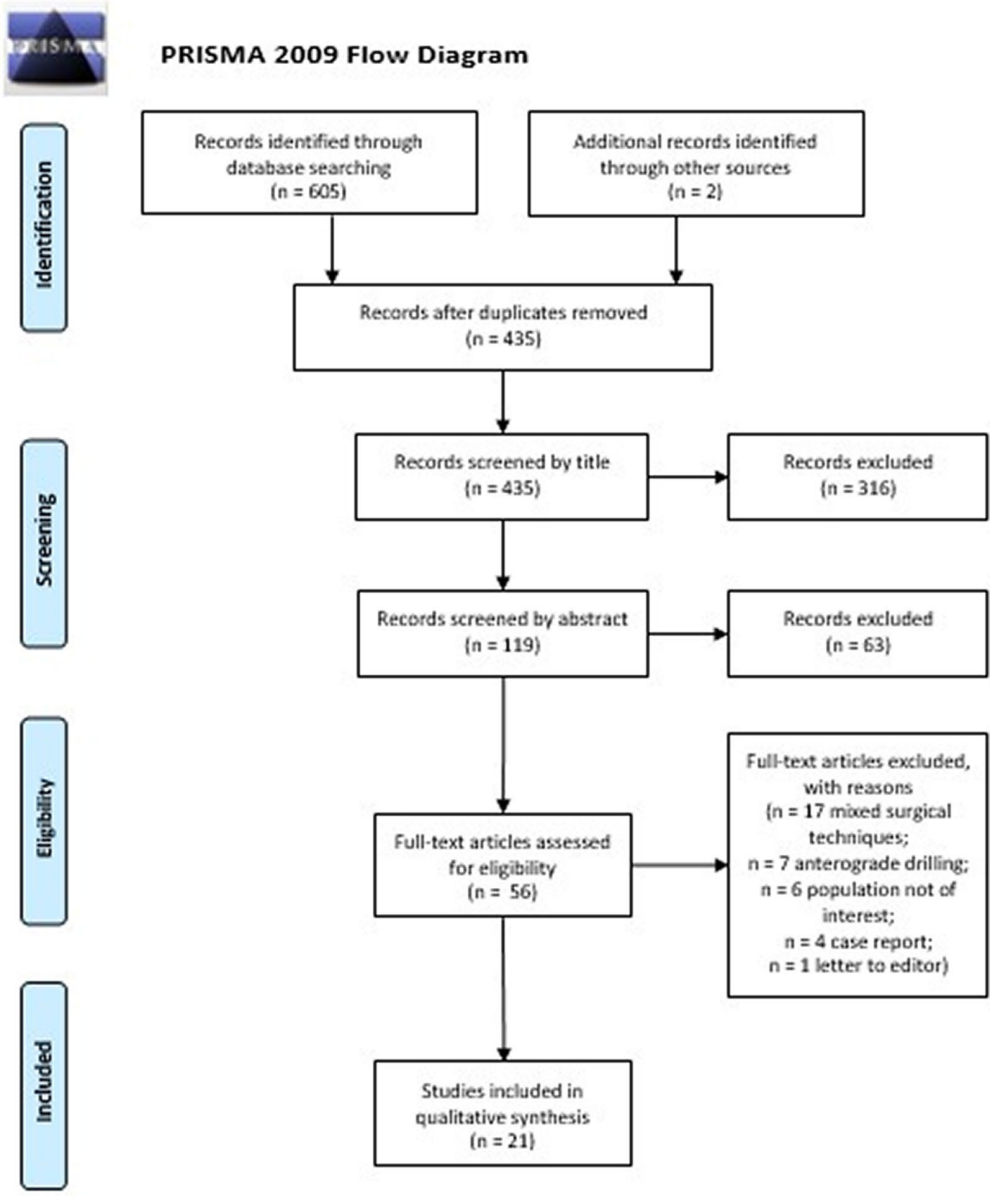
PRISMA flowchart showing the study selection process

A total of 607 articles were identified via database searching and cross-referencing. After duplicate removal, 435 articles were screened by title and abstract, leaving 56 full texts to be assessed for eligibility. Thirty-five articles were excluded because of the following reasons: mixed surgical techniques (*n* = 17); anterograde drilling (*n* = 7); population not of interest (*n* = 6); case report (*n* = 4); letter to the editor (*n* = 1). Twenty-one studies fulfilled the inclusion criteria and were included in the qualitative synthesis. The PRISMA flowchart showing the selection process is presented in Fig. 1.

### Assessment of risk of bias and quality of recommendation

The quality assessment of the included studies, conducted through the ROBINS-I tool for non-randomized studies, is reported in Table 2. Due to the considerable amount of retrospective case series (17 articles, 81%), there was a high likelihood of selection bias. The study's overall risk of bias score was low–moderate, indicating a moderate to good level of methodological quality.

**Table 2:** Risk of bias (ROBINS-I) for the case series

Author, year, reference	Bias due to confounding	Bias in the selection of participants in the study	Bias in the classification of interventions	Bias due to deviation from the intended interventions	Bias due to missing data	Bias in the measurement of outcomes	Bias in the selection of the reported results	Overall
Kono, 2006 [27]	L	L	L	L	M	L	L	L
Kerimaa, 2014 [6]	M	C	L	L	M	L	M	M
Schwartz, 2021 [12]	L	L	M	L	L	L	L	L
Rosenberger, 2006 [13]	M	M	L	L	C	C	S	S
Pritsch, 1986 [14]	M	M	L	L	S	M	C	S
Beck, 2016 [28]	L	M	L	L	M	L	L	M
Yasui, 2014 [15]	L	M	L	L	M	L	L	M
Takao, 2010 [29]	L	L	L	L	L	L	L	L
Ikuta, 2020 [16]	M	M	L	L	L	L	L	M
Anders, 2012 [17]	L	L	L	L	L	L	L	L
Masquijo, 2016 [18]	L	L	L	L	L	L	L	L
Saxena, 2022 [7]	L	M	L	L	M	L	L	M
Flick, 1985 [19]	L	L	M	L	L	M	L	M
Angermann, 1989 [20]	M	S	M	L	M	M	S	S
Taranow, 1999 [21]	L	C	L	L	C	L	L	C
Kelbérine, 1999 [22]	L	C	L	L	M	M	L	S
Bale, 2001 [23]	L	L	L	L	S	L	L	M
Hyer, 2008 [24]	L	S	M	L	L	L	L	M
Geerling, 2009 [25]	L	L	L	L	L	L	L	L
Minokawa, 2020 [26]	L	L	L	L	L	L	L	L
Faldini, 2022 [8]	L	M	M	L	C	L	C	C

^*^L low, *M* moderate, *S* severe, *C* critical

Since most of the included articles were observational case series investigating only patients exposed to the intervention, the quality of evidence was rated as low to very low according to the GRADE framework (Table 3).

**Table 3:** Summary of selected studies

Author, year, reference	Type of study and level of evidence (LOE)	Grade	Number of patients (ankles)	Males:females	Age at surgery (years)	Site of lesion	Size of lesion	Follow-up (months)
Kono, 2006 [27]	Retrospective comparative study—LOE III	Low	11 (11)	7:4	25 (9–47)	Talus 2L 9M		32
Kerimaa, 2014 [6]	Retrospective case series—LOE IV	Low	4 (4)	1:3	20.5 (11–31)	Talus M	14 × 9 x 6 mm	27,6
Schwartz, 2021 [12]	Retrospective case series—LOE IV	Low	7 (7)	1:6	37.6	Talus	8.7 × 9.3 mm	76,2
Rosenberger, 2006 [13]	Retrospective case series—LOE IV	Very low	15 (15)	11:4	34.1 (14–55)	Talus		
Pritsch, 1986 [14]	Retrospective case series—LOE IV	Very low	6 (6)	3:3	30.5 (18–41)	Talus 4L 2M		31,8
Beck, 2016 [28]	Prospective observational study—LOE III	Low	7 (7)	3:4	36 (18–69)	Talus M	14 × 10 x 9 mm	24,1
Yasui, 2014 [15]	Retrospective case series—LOE IV	Low	16 (16)	5:11	25 (14–49)	Talus M		29
Takao, 2010 [29]	Prospective observational study—LOE III	Low	11 (11)	5:6	27.7 (13–48)	Talus + tibia	12.8 mm	48
Ikuta, 2020 [16]	Retrospective case series—LOE IV	Low	8 (8)	5:3	14.9 (11–19)	Talus 7M 1C	9.8 × 6.8 x 3.5	48
Anders, 2012 [17]	Retrospective case series—LOE IV	Low	38 (41)	22:16	33.2 (11–56)	Talus 36M 4L 1C	9 mm	29
Masquijo, 2016 [18]	Retrospective case series—LOE IV	Low	6 (6)	5:1	13 (11–15	Talus 5M 1C		37
Saxena, 2022 [7]	Retrospective comparative study—LOE III	Low	47 (47)			Talus		
Flick, 1985 [19]	Retrospective case series—LOE IV	Low	19 (19)	10:9	28 (9–57)	Talus 7M 12L		25,8
Angermann, 1989 [20]	Retrospective case series—LOE IV	Very low	10 (10)		34 (15–57)	Talus		
Taranow, 1999 [21]	Retrospective case series—LOE IV	Very low	16 (16)		33 (16–44)	Talus M		24
Kelbérine, 1999 [22]	Retrospective case series—LOE IV	Very low	3 (3)		26	Talus		60
Bale, 2001 [23]	Retrospective case series—LOE IV	Low	4 (4)	2:2	23 (18–35)	Talus M		
Hyer, 2008 [24]	Retrospective case series—LOE IV	Low	8 (8)		36 (12–49)	Talus M		24
Geerling, 2009 [25]	Retrospective case series—LOE IV	Low	20 (20)	12:8	35 (19–58)	Talus 16M 4L		25
Minokawa, 2020 [26]	Retrospective case series—LOE IV	Low	6 (8)	4:2	11.1	Talus 4M		22,8
Faldini, 2022 [8]	Retrospective case series—LOE IV	Very low	4 (4)	3:1	41.5 (24–60)	Talus 3M 1L	11.8 mm (9–14)	3

^*^M medial, *L* lateral

### Study characteristics and results of individual studies

The literature concerning RD was mostly constituted by retrospective case series (17 articles, 81%) [6, 8, 12–26], classified as LOE IV. The remaining articles were equally divided into retrospective comparative studies (two articles, 10%) [7, 27] and prospective observational studies (two articles, 10%) [28, 29], both classified as LOE III (Table 3). The majority of the included studies were published in the last 20 years.

A total of 266 patients (271 ankles) were included in this review. When reported, the sex distribution was 99 (54%) males and 83 (46%) females. Mean age at surgery was available in all articles but one [7], and was found to be 28.2 years (range, 9–69). Final follow-up was on average 33 months, and was reported in all articles but four [7, 13, 20, 23].

Most of the osteochondral lesions were in the talus; only Takao et al. included lesions on both the talus and distal tibia [29]. Concerning the osteochondral lesions of the talus, a more detailed localization was provided in 16 articles as follows: 83% (144/174) medial, 15% (27/174) lateral, 2% (3/174) central. Antero-posterior localization was not considered when performing this calculation; therefore, lesions described as posteromedial or anterolateral were counted as medial and lateral, respectively.

Seven articles reported the mean size of the lesions, but in different ways [6, 8, 12, 16, 17, 28, 29]. To compare most of the articles, only the mean length of the lesions was considered; it was found to be 11.4 mm (Table 3).

### Synthesis of results

Among the clinical and radiological parameters used in assessing surgical indication, 15 authors agreed that intact cartilage must be present to make patients eligible for RD [7, 8, 12, 14, 16–27].

The Berndt and Harty classification [30] was considered in four articles: two authors recommended surgical treatment in grades I–III [13, 17], whereas Masquijo and Geerling restricted the indication to only grades I and II [18, 25]. Regarding lesion size, only two authors set a threshold value to consider surgery, which ranged from 100 mm^2^ to 125 mm^2^ [7, 28]. Faldini et al. also extended surgical indication to wide osteochondral lesions with, theoretically, no size limit [8].

Osteochondral lesions were classified by applying different grading systems based on the available radiological imaging. The Berndt and Harty classification on a plain radiograph was used in six studies, with an average grade of 2.2 out of 4 [6, 14, 18, 19, 21, 23]. The Pritsch classification on arthroscopic imaging was used in five studies, with a mean grade of 1.6 [14, 17, 26–28]. Other less common grading systems applied are reported in Table 4.

**Table 4:** Indications and surgical procedure

Author, year, reference	Indications	Classification and grades	Navigation	Adjuvant
Kono, 2006 [27]		Pritsch 0: 2; I: 9 Nelson I	Fluoroscopic	
Kerimaa, 2014 [6]		Berndt and Hardy I and II	MRI	
Schwartz, 2021 [12]	Intact cartilage cap		Fluoroscopic	
Rosenberger, 2006 [13]	Berndt and Harty stages I–III		Computer assisted + fluoroscopic	
Pritsch, 1986 [14]	Intact cartilage cap	Berndt and Hardy II: 1; III: 3; IV: 2		
Beck, 2016 [28]	At least 100 mm^2^ Bristol grade II and partially detached grades III and V	Bristol II: 3; III: 2; V: 2 Pritsch II: 5; II and III: 2	Microvector	PRO-DENSE^®^
Yasui, 2014 [15]		Nelson I: 16 Pritsch I: 16	Fluoroscopic	
Takao, 2010 [29]			Fluoroscopic	Cancellous bone plug
Ikuta, 2020 [16]	Intact cartilage cap	ICRS 0: 3; I: 5 Anderson II: 3; III: 5		
Anders, 2012 [17]	Intact cartilage cap Berndt and Hardy stages I–III	Pritsch I: 12; II: 22; III: 7	Fluoroscopic	Autologous cancellous bone graft
Masquijo, 2016 [18]	Intact cartilage cap Berndt and Harty stages I–II	Berndt and Harty I: 5; II: 1	Fluoroscopic	
Saxena, 2022 [7]	< 125 mm^3^ Intact cartilage cap		Fluoroscopic	Autogenous bone grafting with PRP
Flick, 1985 [19]	Intact cartilage cap	Berndt and Harty II and III: 12; IV: 7	Fluoroscopic	
Angermann, 1989 [20]	Intact cartilage cap		Fluoroscopic	
Taranow, 1999 [21]	Intact cartilage cap	Berndt and Harty IIA: 6; IIB: 2; III: 3; IV: 6	Fluoroscopic	Calcaneal bone graft
Kelbérine, 1999 [22]	Intact cartilage cap			
Bale, 2001 [23]	Intact cartilage cap	Berndt and Harty II: 1; III: 2; IV: 1	Computer assisted + fluoroscopic	
Hyer, 2008 [24]	Intact cartilage cap		Fluoroscopic	Grafton gel^™^
Geerling, 2009 [25]	Intact cartilage cap Berndt and Harty stages I and II	Hepple I: 3; IIa: 10; IIb: 4; V: 3	Computer assisted	
Minokawa, 2020 [26]	Intact cartilage cap	Pritsch II: 7; III: 1	Fluoroscopic	
Faldini, 2022 [8]	Intact cartilage cap Wide osteochondral defects		Fluoroscopic	Hyaluronan scaffold with autologous bone marrow aspirate concentrate and bone graft

The number after the colon in the “Classification and grades” column represents the patients number

^*^PRP platelet-rich plasma

Various evolutions of the original surgical procedures have been described by authors. RD was performed by inserting K-wires retrogradely under different navigation systems. Fluoroscopy was the most extensively used method (13 articles) [7, 8, 12, 15, 17–21, 24, 26, 27]. In terms of fluoro-free surgeries, magnetic resonance imaging (MRI) was used in one article [6], and computer-assisted navigation was used by three authors [13, 23, 25]. Finally, Microvector was used by Beck et al. [28].

Different adjuvants were employed after RD. Cancellous bone grafting was utilized to plug the bone defect by four authors [7, 17, 21, 29]. Saxena et al. also injected platelet-rich plasma onto the bone graft [7]. Faldini et al. retrogradely positioned a hyaluronan scaffold soaked in bone marrow aspirate concentrate and, in addition, filled the talar tunnel with cancellous bone graft [8]. Other injectable solutions were applied alone in a retrograde fashion: Grafton^™^ gel by Berlet et al. [24] and PRO-DENSE^®^ by Beck et al. [28] (Table 4).

In order to compare the clinical outcomes, 12 articles (195 ankles) preoperatively recorded the AOFAS score, and 12 articles (198 ankles) recorded it postoperatively. The mean preoperative value was 64.2 (range, 0–87), which increased postoperatively to 88.8 (range, 48–100) [7, 13, 15–18, 21, 24, 25, 27–29].

Pain assessment was conducted through VAS by six authors (78 ankles), and the results were on average 6.9 and 2.2 before and after surgery, respectively [6, 8, 12, 14, 18, 19].

Post-treatment satisfaction was investigated in 59 patients, and satisfactory results were achieved in a mean of 83% of the patients [12, 14, 18–20, 22, 26] (Table 5).

**Table 5:** Clinical outcomes and complications

Author, year, reference	AOFAS preop	VAS preop	AOFAS postop	VAS postop	Complications	Satisfaction
Kono, 2006 [27]	72.8		97.2		0	
Kerimaa, 2014 [6]		7.5		1.75		
Schwartz, 2021 [12]		7		4.7		85.7% satisfied
Rosenberger, 2006 [13]			88.9 (75–100)			
Pritsch, 1986 [14]					1 progression	3 good; 3 fair; 1 poor
Beck, 2016 [28]	71		90.3			
Yasui, 2014 [15]	73.4 (62–87)	5.5 (4–8)	91.2 (85–100)	0.6 (0–20)		
Takao, 2010 [29]	66 (59–73)		95.8 (90–100)			
Ikuta, 2020 [16]	69.3 (59.6–78.9)		97.1 (93,3–100,9)		0	
Anders, 2012 [17]	47.3	7.5	80.8	3.7	5 ankle swelling 2 minor hypesthesia; 1 delayed wound healing	
Masquijo, 2016 [18]	69 (55–75)	6.2 (4–8)	98 (90–100)	0.3 (0–2)	0	100% satisfied
Saxena, 2022 [7]	76.35		96.08			
	73.13 (+ PRP)		95.63			
Flick, 1985 [19]						79% excellent/good; 21% fair
Angermann, 1989 [20]						85% satisfied
Taranow, 1999 [21]	53.9 (37–75)		82.6 (48–100)		0	
Kelbérine, 1999 [22]					No serious complications	2 excellent; 1 good
Bale, 2001 [23]					0	
Hyer, 2008 [24]	22 (0–41)		56 (52–68)		0	
Geerling, 2009 [25]	76 (68–82)		85 (69–100)			
Minokawa, 2020 [26]						7 good 1 poor
Faldini, 2022 [8]	65 (60–71)	7.75 (7–9)			0	

A few articles registered postoperative complications [8, 14, 16–18, 21–24, 27] (Table 5). In most cases (91%), no complication occurred. Ankle swelling was the most frequently reported complication (five cases), followed by two cases of minor hypoesthesia, one case of delayed wound healing, and one case of progression of the osteochondral lesion.

As Kelbérine et al. merely stated that there was “no serious complication,” it was excluded from this calculation, as some minor complications may have occurred [22].

## Discussion

Results from this review confirmed that RD is a safe, effective, low-morbidity procedure for the treatment of ankle osteochondral lesions. Improvements in postoperative AOFAS and VAS were observed at short/medium-term follow-up. However, some concerns remained regarding the risk of degenerative arthritis over time in those cases with persistent lesions on radiographs. The fact that RD was commonly recommended for small lesions with intact cartilage could have contributed to the favorable outcomes, as these lesions are often easier to treat and may have fewer symptoms compared to larger or more extensive lesions. Regardless of the lesion size, the most common criterion for RD was the presence of a subchondral cyst with intact overlying cartilage [7, 8, 12, 14, 16–26]. Moreover, recent modifications to the original technique allowed the indication to be extended to osteochondral lesions with damaged cartilage, but no long-term results have been provided yet [8].

According to the literature, RD was indicated in the treatment of small lesions (area < 100 mm^2^, depth < 5 mm, diameter < 10 mm) [31] and when defects were difficult to reach through usual arthroscopic portals. The results of this review showed a mean lesion length of 11.4 mm, which was higher than what was recommended. In fact, there was no complete agreement regarding the proper indication for RD, and many surgeons arbitrarily referred to their clinical experience.

Concerning the surgical procedure, some differences were found in the navigation systems, with fluoroscopy being the most commonly used [7, 8, 12, 15, 17–21, 24, 26, 27]. Drawbacks of fluoroscopic guidance included difficulty in osteochondral lesion identification and a lack of 3­D imaging intraoperatively [13, 23, 25]. For this reason, up to 20% of fluoroscopically navigated drilling procedures have been shown to be inaccurate, and a drill misplacement can be found in up to 28% of cases [13, 23, 25]. To overcome these issues, other systems have been proposed, such as drill guide systems [28] and, more recently, navigation systems guided by MRI or computed tomography (CT) or that are computer assisted [13, 23, 25]. Although authors argued that newer guidance systems have advantages in terms of reliable cartilage visualization and the precise treatment of osteochondral lesions, the average procedure time did not demonstrate a speed advantage compared to the standard fluoroscopic technique [6]. Moreover, the higher costs of the procedure should also be considered.

Some modifications to the original surgical technique have been made over the years. The drilling technique has been modified by using a 6-mm or 8-mm drill to allow lesion decompression and better cyst curettage. A wide talar tunnel may also be necessary to insert biological adjuvants [8, 28]. Further variations involved the filling of the drill hole and the defect with different materials. One of the most commonly applied was a cancellous bone graft [7, 17, 21, 29], to which some authors added platelet-rich plasma [7]. Other injectable solutions were employed retrogradely, such as Grafton^™^ gel [21] and PRO-DENSE^®^ [28]. Of particular interest was the retrograde application of a hyaluronan scaffold soaked in bone marrow aspirate concentrate, which combines the advantages of reparative and regenerative techniques [8]. This cartilage-sparing technique derived from RD [8] was developed as an alternative to metal-resurfacing implants [32] and for addressing deeper defects or lesions involving the talar gutter, where thorough osteochondral debridement is necessary.

This systematic review has provided a comprehensive overview of actual indications, surgical techniques, and clinical outcomes of RD, which may be useful to surgeons when choosing among the various treatments of ankle osteochondral lesions. RD is a bone-marrow-stimulating technique which is supposed to induce the production of fibrocartilage at the site of the treated lesion. Since some authors have associated regenerative procedures with standard RD, and it would be highly interesting for future research to evaluate the type of cartilage produced using T2 mapping [2, 8].

When analyzing the results of this review, the nature of the included studies imposes a limitation, as they were mainly retrospective case series and therefore open to selection and detection bias, as shown by the ROBINS-I and GRADE evaluations. Moreover, most of the studies had a small sample size. The mean follow-up was sufficient for assessing postoperative complications but not long enough to evaluate long-term effects. Lastly, postoperative outcomes were only reported through clinical scores, and no radiological outcomes were provided. In addition, different measurements of clinical outcomes and missing data represent some limitations of the study. Therefore, wider samples and are longer follow-up are needed to confirm these early results and to more deeply investigate the effect of RD.

## Conclusions

This systematic review has highlighted the suitability of RD for treating subchondral cysts with intact cartilage or small lesions, with good postoperative satisfaction and symptom relief achieved at middle-term follow-up. Modifications to the technique allowed for broader surgical indications, even in complex cases. RD can be performed with different navigation systems and adjuvants, yielding similar outcomes. However, further research is necessary to assess long-term results.

## Data Availability

The datasets used and/or analyzed during the current study are available from the corresponding author on reasonable request.
